# Learning Reflectance Confocal Microscopy of Melanocytic Skin Lesions through Histopathologic Transversal Sections

**DOI:** 10.1371/journal.pone.0081205

**Published:** 2013-12-05

**Authors:** Juliana Casagrande Tavoloni Braga, Mariana Petaccia Macedo, Clovis Pinto, João Duprat, Maria Dirlei Begnami, Giovanni Pellacani, Gisele Gargantini Rezze

**Affiliations:** 1 From the Cutaneous Oncology Department, A C Camargo Hospital, Sao Paulo, Brazil; 2 From the Anatomy Pathology Department, A C Camargo Hospital, Sao Paulo, Brazil; 3 From the Dermatology Department, University of Modena and Reggio Emilia, Modena, Italy; The University of Queensland, Australia

## Abstract

Histopathologic interpretation of dermoscopic and reflectance confocal microscopy (RCM) features of cutaneous melanoma was timidly carried out using perpendicular histologic sections, which does not mimic the same plane of the image achieved at both techniques (horizontal plane). The aim of this study was to describe the transverse histologic sections research technique and correlate main dermoscopic features characteristic of cutaneous melanoma (atypical network, irregular globules and pseudopods) with RCM and histopathology in perpendicular and transverse sections in order to offer a more precise interpretation of in vivo detectable features. Four melanomas and 2 nevi with different dermoscopic clues have been studied. Lesion areas that showed characteristic dermoscopic features were imaged by dermoscopy and confocal microscopy and directly correlated with histopathology in perpendicular and transverse sections. We presented the possibility to perform transverse sections as a new approach to understand RCM features. Atypical network showed different aspects in the 2 melanomas: in one case it was characterized by pleomorphic malignant melanocytes with tendency to form aggregates, whereas in the other elongated dendritic cells crowded around dermal papillae, some of them forming bridges that resembled the mitochondrial aspect at confocal and histopathology transversal sections. Pigment globules in melanomas and nevi differed for the presence of large atypical cells in the former, and pseudopods showed up as elongated nests protruded toward the periphery of the lesion. Transverse histologic research sections have a consistent dermoscopic and confocal correlate, and it may represent an help in confocal feature interpretation and an advance in improving melanoma diagnosis and knowledge of the biology of melanocytic lesions.

## Introduction

In the last decades development of new techniques, such as dermoscopy, improved melanoma diagnostic accuracy. Dermoscopic diagnosis is based on the identification of characteristic patterns related with specific histopathologic substrates [1], [2].

Recently, in vivo reflectance confocal microscopy (RCM), a noninvasive imaging technique that produces horizontal images of the skin with cellular level resolution from the surface to the papillary dermis, offered the opportunity to detect characteristic histologic features, improving skin tumor diagnosis accuracy [3]. Like dermoscopy, RCM reveals morphologic details of architecture in the en face plane, but, in addition, it provides morphologic information on the cellular level [4]–[7].

The histopathologic interpretation of dermoscopic and RCM findings, using routine perpendicular sections, does not mimic the same plane of the image achieved at both techniques, which produce a horizontal overview of pigmented skin lesions [5], [8]. Therefore, transverse histologic sections could contribute to better characterize the features observed by dermoscopy and RCM. Although this is a research method considered being technically complicated, Rezze et al. have demonstrated that this is a procedure allowing a reliable correlation between dermoscopic features and histopathological findings [9].

In this study we sought to correlate main dermoscopic features characteristic of cutaneous melanoma (atypical network, irregular globules, radial streaming and pseudopods) with RCM and histopathology in perpendicular and transverse sections in order to offer a more precise interpretation of in vivo detectable features.

## Materials and Methods

### Study Population

Four melanomas, with dermoscopic diagnosis by pattern analysis method, have been studied: two of which showing atypical network, one case displaying irregular globules and one case presenting pseudopods. Moreover, two nevi with the benign counterpart of melanoma dermoscopic clues have been included as controls: a junctional nevus with typical network and a compound nevus with regular globules. Melanomas presented in this paper were equal to or larger than 1.0 cm in diameter, with a clear-cut dermoscopic diagnosis based on the previously described pattern analysis method. Patients were recruited at the Department of Cutaneous Oncology of the Brazilian Cancer Hospital A. C. Camargo, São Paulo. All patients signed an informed consent and this study was approved by the A C Camargo Cancer Center Ethics Committee (CEP 1524/11).

#### Instruments

Digital dermoscopy imaging was performed by high-resolution digital dermoscope (DermLite Foto; 3GEN LLC, Dana Point, CA) for all cases. RCM images were acquired by means of near-infrared reflectance confocal laser scanning microscope (Vivascope 1500®; Lucid Inc., Rochester, NY, USA). Instruments and acquisition procedures are described elsewhere [10], [11].

Briefly, the RCM adapter ring was centered onto the lesion area of interest, to ensure a direct correlation among dermoscopic, confocal microscopic, and histopathologic features. A low-resolution dermoscopic camera integrated into Vivascope software was used to allow precise confocal navigation and to look at dermoscopic images (VivaCam®).

Confocal image acquisition included a minimum of 3 mosaics (Vivablock®), corresponding to montages of single high resolution images acquired in an automated series at the same depth, with an area ranging from 4×4 to 8×8 mm at three different depth levels (i.e. intraepidermal, dermal-epidermal junction and superficial dermis). Moreover, series of high-resolution images (capture and stack images) were obtained at different levels from the surface down to the papillary dermis. Each image (1000×1000 pixels) corresponds to a horizontal section at a selected depth with a 500×500 µm field of view with an approximately 1 µm lateral resolution.

After dermoscopic and RCM image acquisition, an ink mark at 1 pole of the specimen was positioned to make its orientation easier.

#### Histopathologic procedure

After the dermoscopic diagnosis of cutaneous melanoma, we selected one of the following features for biopsy: atypical network, irregular globules, radial streaming or pseudopods. To avoid interference with Breslow’s index, selected features were always restricted to the epidermis. The site of the interest area was marked with a silk suture. After the excisional biopsy, the tissue was formalin fixed and paraffin embedded.

Afterward the marked area was removed using a 4 mm punch biopsy and the remaining tissue sample was used for routine diagnosis according to the Pathology Department’s protocol. Before the punch sample being processed, it is anticipated the final report of the pathologist. If there is no conflict as to the final diagnosis, the biopsy sample was sliced into two halves: one of the fragments had perpendicular sections and the other had serial microscopic transverse sections from the epidermis toward the dermis, adapted from Headington [12].

In our protocol extra paraffin is added to avoid wearing out the *stratum corneum* with the first slices. Consecutive 5 µm thick sections are taken from the tissue block starting from the outer layer of the *stratum corneum* to the dermis, and then stained with Haematoxylin & Eosin (H&E) for correlation with the confocal findings. The other half of the biopsy was conventionally sectioned and stained. Breslow’s index is revised in this fragment just to ensure that it was equal or thinner than the remaining excisional specimen.

Histopathologic pictures were acquired using ScanScope Digital Slide Scanner (Aperio, Vista, CA, USA).

### Image Description

Dermoscopic image description of each pattern was performed according to the definition of the literature [1], [2], [8]. The RCM images were described using the terms previously proposed and recently summarized in a consensus terminology glossary [13]–[21]. Histopathologic description was performed and it was mainly based on the presence and distribution of typical/atypical melanocytes and pigmented keratinocytes within epidermis and dermal-epidermal junction (DEJ), and melanophages, inflammatory infiltrate and vessels within the dermis.

## Results

Confocal aspects and their histologic substrates (perpendicular and transverse sections) for each dermoscopic feature are summarized in the table 1. In none of the cases the procedure interfered with Breslow thickness determination.

**Table 1:** Summary of dermoscopic features, reflectance confocal microscopy and histopathologic aspects.

	Dermoscopic feature	Confocal aspects	Transverse section	Perpendicular section
**Case 1**	Slightly pigmentednetwork	Irregular and dishomogeneous dermalpapillae. Dendritic cells bulge from theepidermis toward the dermis forming“bridges” called mitochondria likestructures.	An increased number of atypical melanocytes arranged around the dermal papillae. The atypical melanocytes protrude into thedermal papillae forming bridgesthus confirming the mitochondrialstructures in RCM.	Disarrangement of the rete ridge and the increased number of atypical melanocytes. It is not possible to see the mitochondrial structure in this view.
**Case 2**	Broadened pigmentednetwork	Irregular and dishomogeneous dermalpapillae. Demarcated and non-demarcated rings were separated byloosely thick interpappilary spaces.	Predominance of atypical melanocytes, isolated or in nests, enlarging the interpapillary spaces.	Disarrangement of the rete ridge and the increased number of atypical melanocytes in the epidermis.
**Case 3**	Typical network	Rings of bright polygonal cellssurrounding roundish to oval darkareas corresponding to dermalpapillae at DEJ. The papillaelacked cytologic atypia.	An increased number of isolated melanocytes are arranged aroundthe dermal papillae and thereare nevus cells nests within theepidermis. The interpapillaryspaces are preserved.	Elongated rete ridges and presence of single and nests of nevus cells in the DEJ
**Case 4**	Irregular globules	Irregularly shaped clusters with cellsthat are nonhomogeneous inmorphologic features and reflectivity.Dense cell clusters consisted of largehomogeneous polygonal cells tightlyaggregated in a roundish regular structure.	Large amount of atypicalmelanocytes predominantlyin nests with variablesize and shape within theepidermis and the dermis.	Compact aggregates of atypical melanocytes with a slight intercellular discohesion, variable in size and shape, predominantly distributed at the DEJ and in the papillary dermis.
**Case 5**	Regular globules	Compact aggregates with sharpmargin of large polygonal cellssimilar in morphologic featuresand reflectivity, forming polyhedralstructures.	Mostly singly nevocytes aroundthe papillae (at the DEJ) anddense nests composed of nevuscells within the dermis surroundedby a narrow band of epidermis.	Singly nevus cells at the DEJ and well-circumscribed melanocytic nests composed of typical, large and monomorphous nevocytes, disposed in an organized manner within the dermis.
**Case 6**	Pseudopods	Compact aggregates of atypical cellsare distributed in a linear arrangementtoward the periphery with a densenest at the extremity, similar aglobulelike structure within theepidermis.	Nests of atypical cells are arrangedin a linear manner throughoutthe periphery of the lesion.	Nests of atypical melanocytes are distributed contiguously toward the periphery along the DEJ.

### Pigment Network

There were 3 cases presenting pigmented network on dermoscopy. Two melanomas were characterized by atypical network, whereas a nevus showed a typical network.

**Figure 1:**
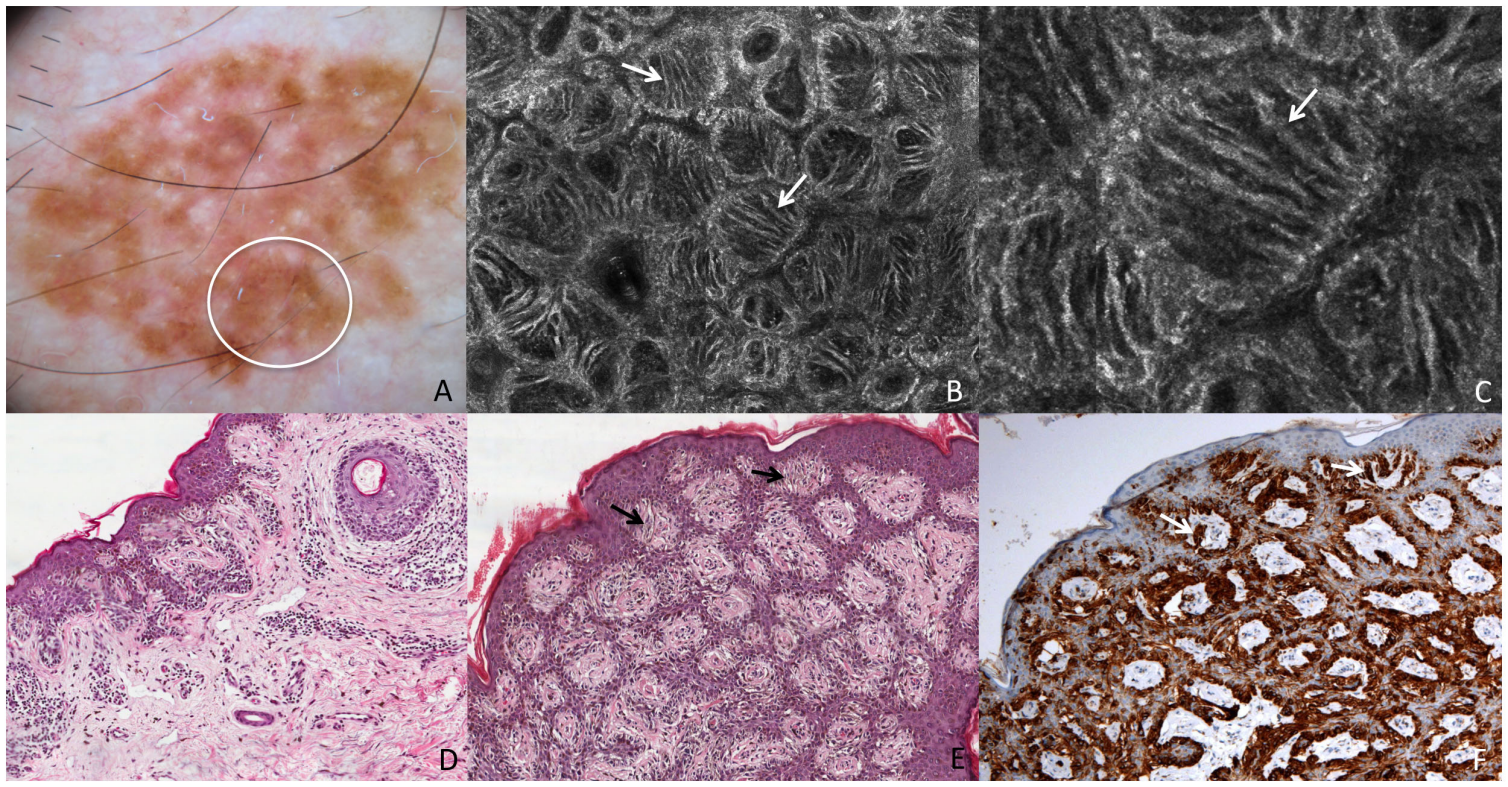
Superficial spreading melanoma *in situ*. This lesion shows on dermoscopy (A) a slightly pigmented network (white circle corresponds to the punch area). RCM mosaic image (B, 1×1 mm) at the level of the DEJ shows irregular and dishomogeneous dermal papillae with dendritic cells (white arrows). RCM individual image (C, 0,5×0,5 mm) at the level of the DEJ shows dendritic cells forming “bridges” called mitochondria-like structures (white arrow). Perpendicular section (D) shows disarrangement of the rete ridge and the increased number of atypical melanocytes. Transverse section (E, HE staining) shows atypical melanocytes protruding into the dermal papillae forming bridges (black arrows). Transverse section (F, Melan-A staining) shows cells positive for Melan-A protruding into the dermal papillae (white arrows).

**Figure 2:**
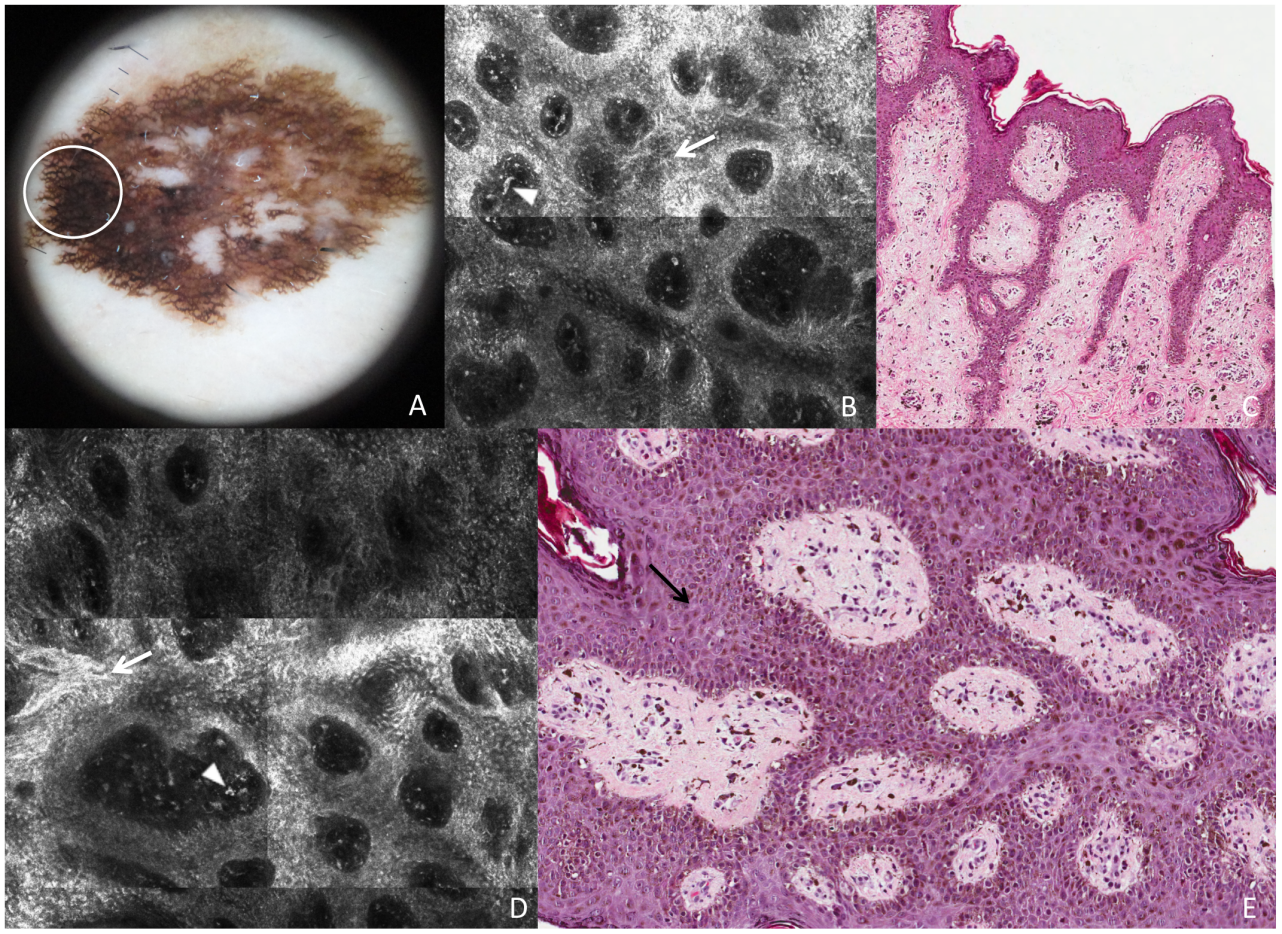
Superficial spreading melanoma *in situ*. This lesion shows on dermoscopy (A) a broadened pigmented network (white circle corresponds to the punch area). RCM mosaic images (B and D, 1×1 mm) at the level of the DEJ show demarcated and non-demarcated rings separated by loosely thick interpappilary spaces (white arrows) and some plump bright cells and bright dots are visible within dermal papillae (arrowheads). Perpendicular section (C) shows disarrangement of the rete ridge and the increased number of atypical melanocytes in the epidermis. Transverse section (E) shows predominance of atypical melanocytes, isolated or in nests, enlarging the interpapillary spaces (black arrow).

Concerning the two melanomas with atypical network, one was showing a tiny thin slightly pigmented presented network (Figure 1A). Melanoma diagnosis was suspected on a clinical base since the lesion as a solitary large mole with history of slow growth over years. The other lesion displayed an irregularly broadened pigmented network (Figure 2A). When using RCM, the atypical pigment network corresponded to a meshwork pattern [22] with irregular and dishomogeneous dermal papillae.

In correspondence of the tiny thin network pattern, a dendritic cell proliferation at the DEJ was observed in RCM. In some instances, some dendritic elongated cells were bulging from the epidermis into the papillae originating “bridges” apparently within the papillary dermis (Figures 1B and C). These figures resemble mitochondria like structures in electron microscopy. The dendritic cell proliferation in the basal layer (accounting for “atypical cells at the DEJ” according with the RCM-score definition) resulted in a positive major criterion for melanoma diagnosis, though the total score was less than the optimal threshold (3 points) for melanoma diagnosis^18^. Perpendicular histologic sections revealed a disarrangement of the rete ridge and increased number of atypical melanocytes in single units and small nests at the DEJ, describing an *in situ* melanoma (Figure 1D). On the other hand, transverse histologic sections revealed an increased number of atypical melanocytes arranged around the dermal papillae. Some of these melanocytes seems to protrude into the interior of the dermal papillae forming bridges that resemble the mitochondrial structures in RCM (Figure 1E and F). RCM study of the broadened atypical pigmented network (Figure 2A) showed irregular enlarged meshes constituted by a pleomorphic (both roundish and dendritic) melanocytic proliferation (Figures 2B and D). Papillary contours are well outlined, but the overall architecture is irregular, with unevenly enlarged meshes corresponding to the broadened network lines as evaluated upon dermoscopy. Some plump bright cells and bright dots were visible within dermal papillae (Figures 2B and D). Transverse histologic sections revealed the exact RCM outlines, with meshes filled of a proliferation of pleomorphic malignant melanocytes with some tendency to aggregates into nests, and inflammatory infiltrate within dermal papillae (Figure 2E). Perpendicular sections showed elongated cristae with a junctional proliferation of atypical melanocytes (Figure 2C).

**Figure 3:**
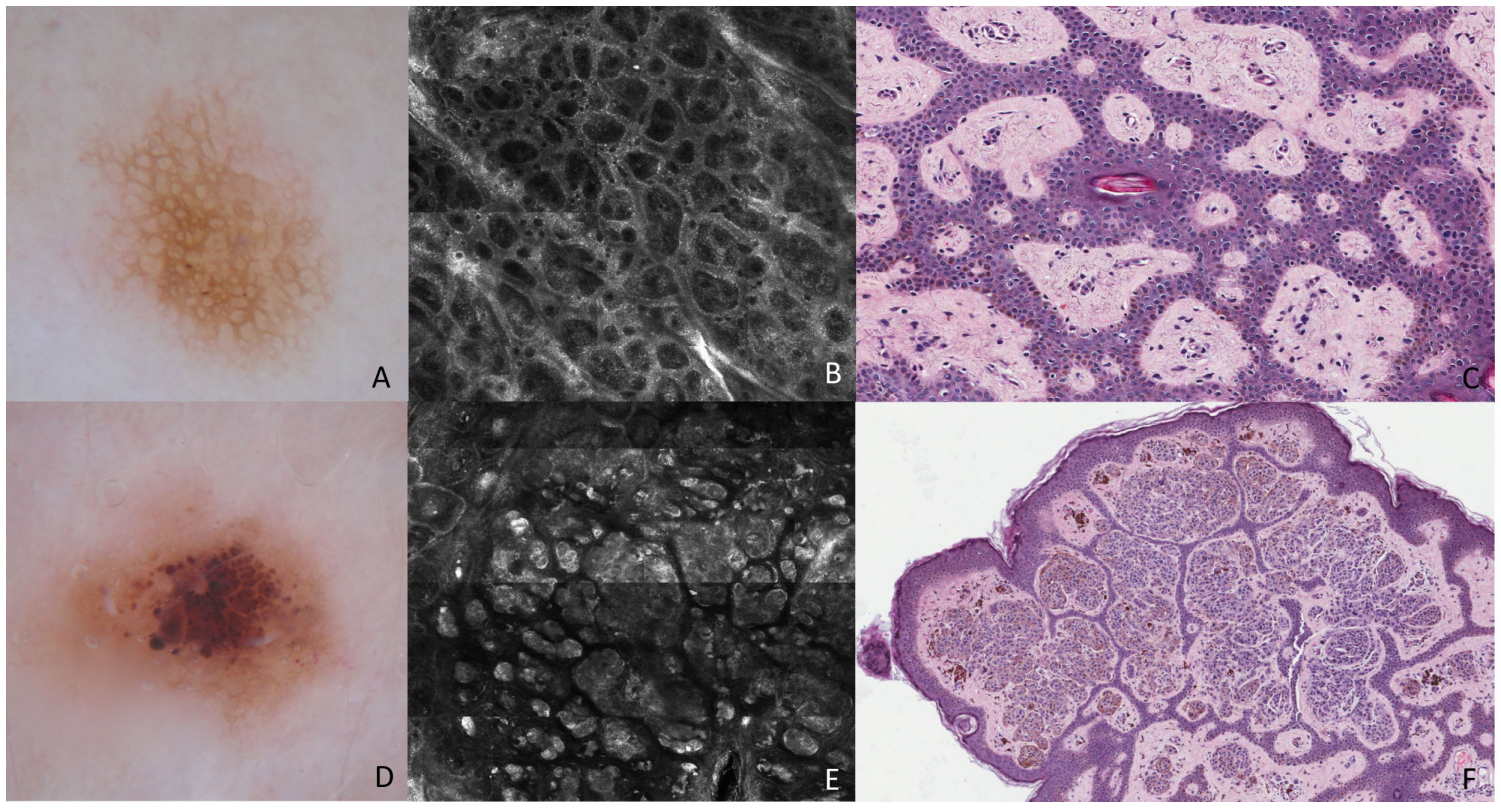
Melanocytic Nevi. These lesions show typical network (A) and regular globules (D), on dermoscopy. RCM mosaic image (B, 1×1 mm) at the level of the DEJ shows rings of bright polygonal cells surrounding roundish to oval dark areas corresponding to dermal papillae at DEJ. Transverse section (C) shows isolated melanocytes arranged around the dermal papillae and there are nevus cells nests within the epidermis. RCM mosaic image (E, 1,5×1,5 mm) at the level of the DEJ and dermis shows compact aggregates of large polygonal cells similar in morphologic features and reflectivity, forming polyhedral structures. Transverse section (F) shows dense nests composed of nevus cells within the dermis surrounded by a narrow band of epidermis.

Concerning typical network (Figure 3A), on RCM it was characterized by rings of bright polygonal cells (small melanocytes and melanin-rich keratinocytes) surrounding roundish to oval dark areas corresponding to dermal papillae at dermoepidermal junction. Rings were clearly outlining the papillary contours and were separated by a thin, structureless, slightly refractive space that sharply contrasted with the dark background (edged papillae) (Figure 3B). No atypical cells were detectable. Routine histologic analysis revealed elongated rete ridges and single cells and few small nests of nevus cells at DEJ. In the transverse histologic sections, an increased number of isolated melanocytes were arranged around the dermal papillae that accounted for the higher brightness of the cells within the rings at RCM examination. (Figure 3C).

### Pigment Globules and Peripheral Structures (Pseudopods)

**Figure 4:**
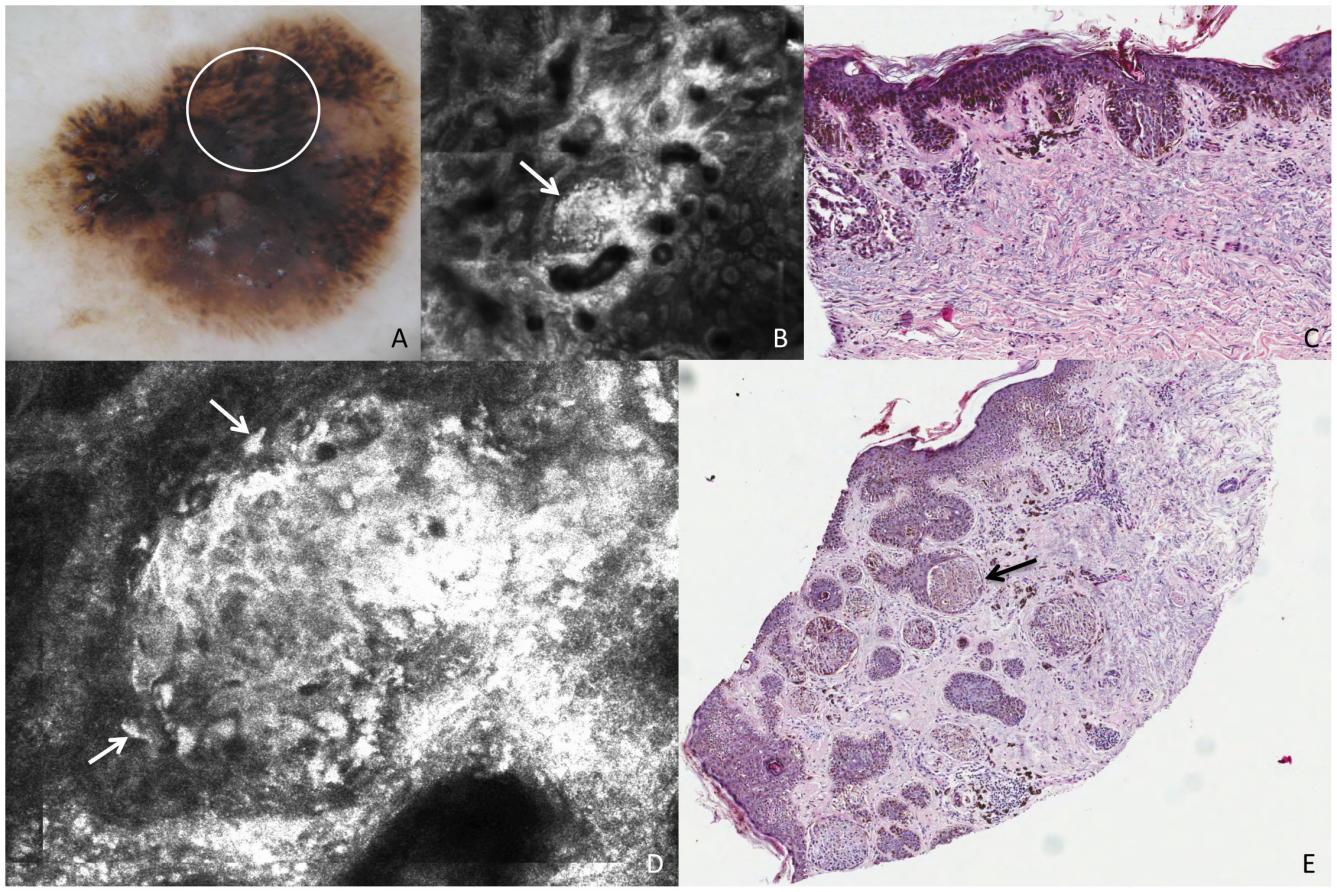
Superficial spreading melanoma, Breslow thickness 0,8 mm. This lesion shows on dermoscopy (A) irregular globules (white circle). RCM mosaic image (B, 1,5×1,5 mm) at the level of the DEJ shows irregularly shaped clusters (white arrow). Perpendicular section (C) shows compact aggregates of atypical melanocytes with a slight intercellular discohesion. RCM individual image (D, 0,5×0,5 mm) at the level of the DEJ shows a cluster with cells that are nonhomogeneous in morphologic features and reflectivity (white arrows). Transverse section (E) shows large amount of atypical melanocytes predominantly in nests with variable size and shape within the epidermis and the dermis. The black arrow points to the nest that makes the exact correlation with confocal image.

The 2 melanomas and the nevus included in the study were characterized by large clusters of bright cells forming nests at RCM examination, regardless for globules and pseudopods. Pigment globules in the melanoma (Figure 4A) showed up as irregularly shaped compact clusters of large atypical cells, non homogeneous in shape and reflectivity (Figure 4D). These dense cell clusters were predominantly bulging within the dermal papillae, connected with the DEJ on one side (Figure 4B).

On perpendicular histologic analysis, atypical globules made of dense clusters of nonhomogeneous cells appeared as compact aggregates of pleomorphic melanocytes with a slight intercellular discohesion, variable in size and shape, predominantly distributed at the DEJ and in the papillary dermis (Figure 4C). Transverse histologic sections revealed large amount of atypical melanocytes predominantly in nests within the epidermis and the dermis related to the globules on dermoscopy and RCM (Figure 4E). Occasionally the nests within the epidermis were confluent, corresponding to broadened network meshes intermingled with the globular aspect visible on dermoscopy, and frequently connected with epidermis.

**Figure 5:**
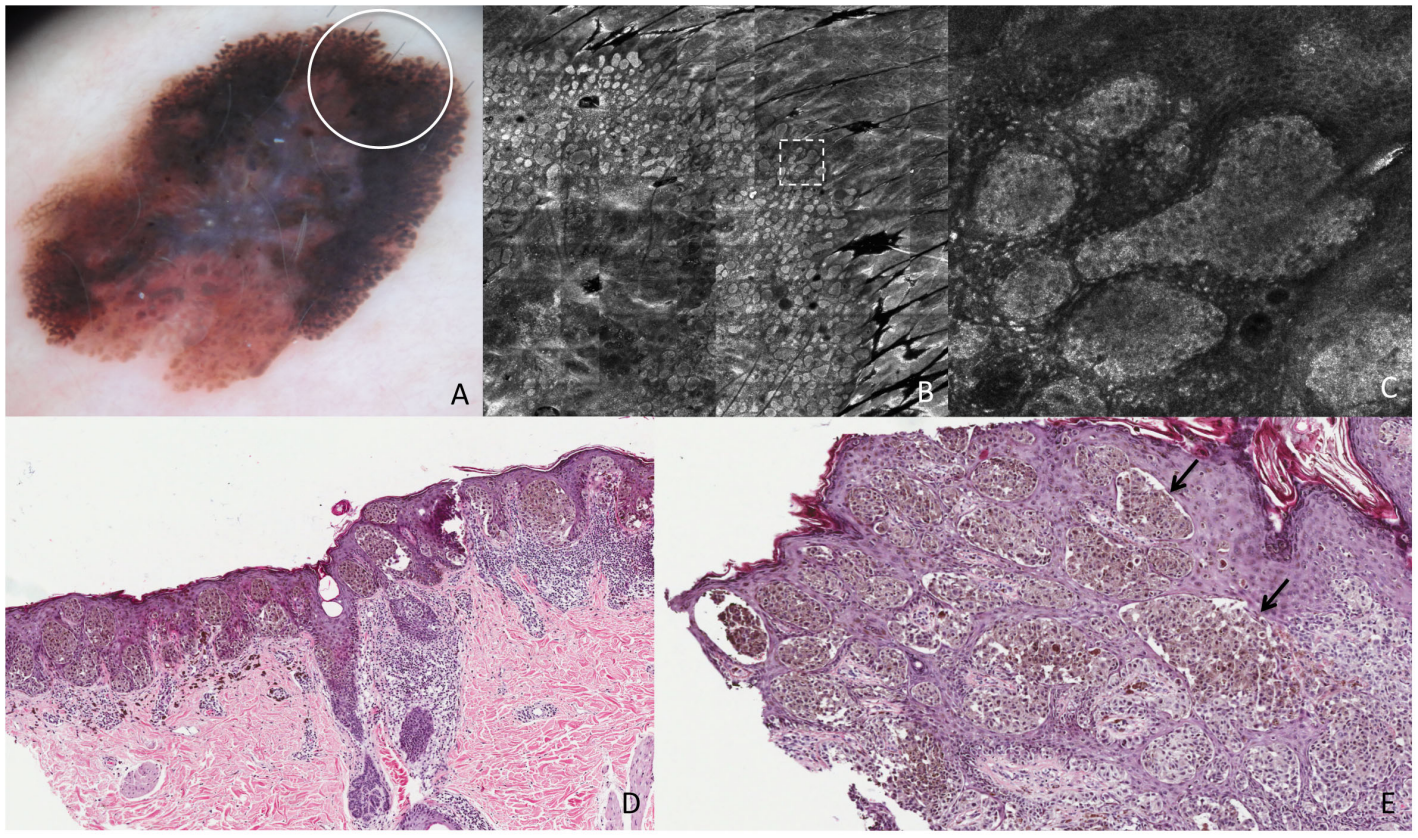
Superficial spreading melanoma, Breslow thickness 0,79 mm. This lesion shows on dermoscopy (A) pseudopods (white circle corresponds to the punch area). RCM mosaic image (B, 3×3 mm) at the level of the DEJ shows compact aggregates of atypical cells distributed in a linear arrangement toward the periphery with a dense nest at the extremity (the area inside the dashed square is represented in figure C). RCM individual image (C, 0,5×0,5 mm) at the level of the DEJ shows a pseudopod in detail, characterized by elongated, dense and bright peripheral aggregate. Perpendicular section (D) shows nests of atypical melanocytes distributed contiguously toward the periphery along the DEJ. Transverse section (E) shows nests of atypical cells arranged in a linear manner throughout the periphery of the lesion (black arrows).

Similarly, pseudopods (Figure 5A) corresponded to clusters of melanocytes, connected with the epidermis, arranged in elongated parallel strands with club-shaped bulges projected toward the periphery. On the internal side, round to oval compact nests with a similar cellular texture were visible at RCM and corresponded to pigment globules upon dermoscopy (Figure 5B). These nests (both peripheral and internal ones) were composed of cell aggregates with ill-defined cellular demarcations and heterogeneous brightness, but they were lacking an evident cytologic atypia (Figure 5C). On perpendicular histologic analysis, malignant melanocytes were arranged predominantly in nests at the epidermis and the DEJ (Figure 5D). Upon traditional histopathologic sections peripheral nests did not differ from the internal ones. In the transverse histologic sections of the DEJ, nests of atypical cells were arranged in a linear manner throughout the periphery of the lesion, and, differently from perpendicular sections, differences of shape were visible between peripheral and internal nests (Figure 5E).

Concerning the globular nevus, an accurate correspondence in shape was observed between brown globules on dermoscopy (Figure 3D) and the dense melanocytic clusters on RCM (Figure 3E), appearing as compact aggregates with sharp margin of large polygonal cells similar in morphologic features and reflectivity, forming polyhedral structures (dense clusters). Routine histologic examination revealed a predominance of well-circumscribed melanocytic nests and cords of typical, monomorphous nevocytes, mainly located within the dermal papillae, and separated by thin epidermal cristae. Transverse histologic sections revealed mostly large dense nests composed of large cohesive nevus cells within the dermis surrounded by a narrow band of epidermis (Figure 3F).

## Discussion

RCM is a noninvasive imaging technique with cellular resolution, offering the opportunity to detect characteristic histologic features in vivo and it has enhanced our ability to assess skin tumors. The fact that RCM evaluates the tissue in the horizontal plane as dermoscopy, and presents high magnification with a cellular-level resolution, such as histopathology, suggests that this technique is a research tool for an excellent correlation with both these methods [5]–[7]. The histologic correspondence of some confocal features has been demonstrated, [4], [14]–[17], [19] although numerous patterns still have to be clearly defined. The increasing interest in using RCM in specialized skin cancer centers derives from the possibility of having a more accurate presurgical diagnosis for different skin tumors, resulting in demonstrated improvement in diagnostic accuracy, especially for melanocytic lesions, also with respect to dermoscopy [3], [20], [22]. Interpretation of dermoscopic and RCM features of cutaneous melanoma is based on histologic description of perpendicular sections of the lesions that does not reflect the overview achieved by both techniques. This paper sought to describe the value of transverse histologic sections as a tool to better characterize the structures observed either in dermoscopy and in confocal microscopy. We demonstrated that transverse histologic sections have a strong and consistent dermoscopic and confocal correlate, and it may be useful for a more accurate interpretation of confocal and dermoscopic alterations, as well as for detection of new diagnostic findings related with specific histopathologic substrates. Furthermore, the possibility of recognizing in vivo cytologic patterns and following them up over time may help to identify MM precursors and to understand the biology of melanocytic lesions [23].

One of the most important dermoscopic criteria in the diagnosis of pigmented skin lesions is represented by the pigmented network [2], [24]–[26]. The dermoscopic appearance of a network has been correlated to histopathological findings; the thicker pigmented rete ridges correspond to the dark brown lines and the thinner pigmented suprapapillary plates correspond to the tan holes [27]. Alterations of network line width and hole size are frequently observed in MMs [16]. On RCM, the atypical network with slightly pigmented lines showed the presence of elongated and spindle cells around and crossing the dermal papillae. This finding is observable in confocal and in transversal sections only, and could not be seen in perpendicular ones. Moreover, it was related with a tiny light pigmented network, which is not different from a slightly irregular network frequently visible in benign lesions. This melanoma represents an exception from the usual presentation of melanoma in dermoscopy and RCM, lacking atypical network and other dermoscopic clues, as well as round pagetoid cells, striking DEJ disarrangement and round/pleomorphic atypical cells in single units and in clusters at the DEJ upon RCM. However, this peculiar pattern corresponded to proliferation of malignant melanocytes mainly in single units at the DEJ, thus accounting for being a relevant diagnostic clue, also in consideration of its evident difference from the RCM substrate in typical pigment network in the nevus.

The broadened pigmented network presented more rounded and polygonal cells than spindle and elongated cells mainly enlarging the rete ridges. We believe the existence of elongated and spindle cells leads to the papillae non-edged in RCM. Thus, we could observe the cytologic and architectural features in the transverse sections were more consistent, showing an exact correlation to dermoscopic pigment network and its corresponding confocal feature.

Dermoscopic globules in nevus and melanoma can be differentiated through cytologic atypia by means of RCM. RCM cell clusters correspond to histopathologic melanocytic nests. Dense clusters of monomorphous cells not connected with epidermis but surrounded by the epithelium characterized the nevus. Unlikely, striking cellular atypia was observable in the nests of the melanoma. Similarly, in transversals we could observe clearly the epithelium surrounding the nests of nevocytes in the nevus and the nests of atypical melanocytes in the melanoma, as they appear in RCM.

Interestingly, pseudopods in melanoma did not show striking cytological pleomorphism at RCM. In histopathology they correspond to confluent nests of pigmented melanoma cells at the periphery of the lesion, in agreement with the concept that these dermoscopic features are specific for the radial growth phase of melanoma [28]. In our case, melanocytic nests at the periphery of the lesion were constituted by medium-sized and quite monomorphic melanoma cells, showing only nuclear striking pleomorphism.

To sum up, from our study we showed the possibility to distinguish the benign or malignant nature of a dermoscopic pattern through its cytological analysis as it can be achieved by in vivo RCM. Histopathologic transversal sections helped us to correlate peculiar RCM findings with their substrate. Moreover, in case of pseudopods a striking RCM cytological pleomorphism does not represent a clue for melanoma diagnosis, since nuclear atypia in absence of cell morphology pleomorphism is not evaluable through RCM.

Last, we presented in our study the possibility to easily, safely and reliably perform transverse sections as a new approach to study and understand RCM feature meaning. Considering the disagreement among pathologists on the diagnosis of MM versus benign melanocytic lesion and the less-than-ideal reproducibility of some histologic features of dysplastic nevi and MM, we may state that the identified RCM features and its direct correlation with transverse sections can help the evaluation of melanocytic lesions by both dermatologists and pathologists, [29] allowing for analysis of the tissue correlates of specific dermoscopic structures [6], [21].

Combination of dermoscopy, RCM and transverse histologic sections may represent an advance in melanoma diagnosis and knowledge on the biology of melanocytic lesions.
